# The Early Diagnosis of Cancer of the Rectum

**Published:** 1894-12-01

**Authors:** 


### The Early Diagnosis of Cancer of the Rectum.

However disagreeable it may be either to patient or doctor, physical examination should be the rule in diseases of the rectum. Time ago it did not matter. If a man had cancer of the rectum the longer he could be kept in ignorance of his sad fate the better, and the best way to keep him in ignorance was for the doctor to remain in darkness also. Now, however, things are on an entirely different footing. The fact that cancer of the rectum can be successfully removed, in even a small percentage of cases, throws on those medical men who fail to diagnose it early a very serious responsibility; for it is only in the early stages, before it has infected the surrounding tissues, that complete removal is possible. Early examination then is necessary, and this is sometimes a difficulty. In hospital practices it is simple enough, but matters are not so easy when the visit is made in a drawing-room, or in an office, or in a cottage with a crowd of gaping children, and maybe neighbours, looking on. An examination under such circumstances involves considerable trouble, its necessity is often vigorously debated, and a special appointment may have to be made. Yet in view of the fact that cancer of the rectum can be successfully removed, if taken sufficiently early, nothing can relieve the medical attendant of the obligation of discovering it at the earliest possible moment. Unfortunately, the general symptoms are inconclusive until considerable progress has been made. Pain is probably the most constant, although often not marked in the early stages. Its situation varies; generally it is in the rectum, but it may, from implication of the sacral plexus, extend down the thighs, simulating sciatica. Constipation is in some cases very marked. Passage of blood and mucus is common, but in the early stages there may be only a little bleeding after a hard stool, and, as the pain also may be chiefly observed under similar circumstances, a risk is run of confounding the disease with fissure of the anus. In fact, from the irritation there may actually be an eczematous condition round the anus, leading to a fissure, which may on mere inspection appear sufficient to explain the symptoms unless a complete examination be made. Diarrhœa alternating with constipation is also a not infrequent early sign, and it cannot be too strongly insisted on that an examination should be made whenever either diarrhœa or constipation are persistent, especially if complicated with the slightest discharge. The term piles covers many ailments and is responsible for many false diagnoses.

In an examination, naturally the first question is as to the nature of the disease, but in regard to treatment several other points have to be observed; specially is it necessary to note how far upwards the growth extends, and how deeply it infiltrates the tissues. The size of the growth, and the amount of the circumference of the bowel implicated, are matters of importance; but the main points favouring the possibility of removal are that there shall be healthy bowel above and that the disease shall not have passed through and beyond the walls of the rectum. The mobility of the bowel and the growth must then be ascertained, and search must be made in the hollow of the sacrum for any signs of enlarged glands in that locality.

Into the more special questions regarding the sort of operation which should be done, we do not here propose to enter. The point on which we wish to insist is that cancer of the rectum no longer remains one of the hopeless diseases which must be left to nature, and the diagnosis of which is therefore unimportant. The experience of recent years shows that in a certain proportion of cases in which it has been removed no recurrence has taken place after such a time as to justify the belief that cure has resulted, and that even in less successful cases a considerable respite has been obtained. But everything hinges on early diagnosis, and that depends on early examination.

